# A Call for Updates to Hormone Therapy Guidelines for Gender-Diverse Adults Assigned Male at Birth

**DOI:** 10.7759/cureus.62262

**Published:** 2024-06-12

**Authors:** Reema Patel, Stanley Korenman, Amy Weimer, Shira Grock

**Affiliations:** 1 Endocrinology, Diabetes and Metabolism, Veterans Affairs Greater Los Angeles Healthcare System, Los Angeles, USA; 2 Endocrinology, Diabetes and Metabolism, University of California Los Angeles David Geffen School of Medicine, Los Angeles, USA; 3 General Internal Medicine, University of California Los Angeles David Geffen School of Medicine, Los Angeles, USA

**Keywords:** anti-androgen, transgender, hormone therapy, gender incongruence, estradiol

## Abstract

Gender-affirming hormone therapy for assigned male at birth (AMAB) individuals with gender incongruence typically consists of estradiol with or without an anti-androgen to achieve physical changes and psychological benefits. However, prescribed hormone regimens vary considerably, and high-quality research in this area is extremely limited. Additional evidence-based research evaluating patient-reported outcome measures (PROMs) is needed to fill current knowledge gaps and create a personalized therapeutic approach for AMAB individuals. This editorial provides a critical description of current treatment options, discusses their variability, reviews some discrepancies in guideline-based dosing recommendations, and recommends areas for further study.

## Editorial

Many assigned male at birth (AMAB) individuals with gender incongruence seek treatment with exogenous estrogen therapy to achieve desired physical and emotional changes. Standard feminizing therapy, according to various professional organizations, includes estradiol in combination with an anti-androgen [1, 2]. However, the data supporting currently recommended regimens are limited and often based on relatively weak justifications.

Estrogen 

Estradiol is administered via oral or sublingual tablets, patches, gels, or injections. Oral estradiol can increase the risk of thrombosis through the release of procoagulant factors during hepatic first-pass metabolism, and non-oral options can be utilized to reduce the risk of venous thromboembolism (VTE) [3]. Estradiol patches and gels likely have the lowest risk of VTE; however, they are formulated for post-menopausal cis women, and the application of numerous patches or large volumes of gel can be burdensome for some gender-diverse individuals who require higher doses. Injectable, and possibly sublingual estradiol, may also offer lower VTE risk compared to oral estradiol; however, further studies are needed.

The current guideline-based dosing recommendations for estradiol vary considerably, which is problematic for clinicians and patients who rely on guidelines to initiate treatment. Most notably, the conversion rates between parenteral estradiol valerate and estradiol cypionate vary drastically between the UCSF Guidelines for the Primary and Gender-Affirming Care of Transgender and Gender Nonbinary People (UCSF Guidelines) and The Endocrine Society Clinical Practice Guidelines for Endocrine Treatment of Gender-Dysphoric/Gender-Incongruent Persons (the Endocrine Society Guidelines). The UCSF Guidelines indicate the conversion between estradiol valerate and cypionate to be as high as a 4:1 ratio [2], while the Endocrine Society Guidelines provide no dosing differentiations [1]. Herndon and colleagues demonstrated that the conversion between estradiol cypionate and estradiol valerate is closer to 1:1 [4]. Further equivalence studies are needed to clarify ideal dosing conversions.

The Endocrine Society Guidelines recommend titrating estradiol to 100-200 pg/mL [1]. The UCSF Guidelines recommend 2-4 mg daily as the starting dose for oral estradiol and 5 mg weekly for parenteral estradiol valerate [2]. The Endocrine Society Guidelines suggest oral estradiol 2-6 mg daily and parenteral estradiol 2-10 mg weekly [1]. However, Chantrapanichkul et al. found that intramuscular injections of estradiol valerate greater than 5 mg weekly led to mean estradiol concentrations well above 200 pg/mL, while 4-5 mg of oral estradiol daily only led to minimum desired concentrations [5]. Similarly, Herndon et al. found that subcutaneous estradiol at a median dose of 3.75 mg per week led to a median estradiol level of 196 pg/mL [4]. Thus, current guideline-based dosing may lead providers to choose doses of injectable estradiol that would result in supratherapeutic serum estradiol levels. In light of these recent publications, it is clear that guideline-based dosing for estradiol needs updating. In our clinical experience, parenteral estradiol valerate at doses of 2-4 mg weekly typically leads to physiologic estradiol levels. Estradiol cypionate should likely be dosed in a 1:1 ratio with estradiol valerate until future data are obtained.

Lastly, while estradiol valerate and cypionate are only FDA-approved for intramuscular administration, many patients prefer subcutaneous administration. There are small studies that suggest the pharmacokinetics of intramuscular and subcutaneous estradiol are similar [4]. While the UCSF Guidelines comment on the use of subcutaneous estradiol, other guidelines should be updated to include this option for patients [2].

Anti-androgens 

While the utility of estrogen for AMAB individuals is well established, the usefulness of anti-androgens remains less certain. Estradiol lowers testosterone by negative feedback on the hypothalamic-pituitary-gonadal (HPG) axis. While some studies suggest that estrogen alone may not fully suppress testosterone levels into the cis female range, other studies have shown that 70% of patients achieve hormone goals on oral estradiol 4 mg daily or more [6, 7]. Practitioners may use anti-androgens, including spironolactone, finasteride, bicalutamide, cyproterone acetate, or gonadotropin-releasing hormone agonists, in addition to estrogen to block testosterone action or lower testosterone production. However, there is little evidence-based guidance on their effectiveness or how to select anti-androgens. For individuals with cis female range testosterone levels on estradiol monotherapy, androgen blockade could lead to sub-physiologic action of testosterone. While data are mixed, low testosterone in cis women has been associated with low sexual desire and, less consistently, depression [8]. To our knowledge, whether low testosterone causes similar effects in the gender-diverse population has not been studied.

Spironolactone is the most utilized anti-androgen in the United States. It is a competitive inhibitor at the androgen receptor; however, whether spironolactone lowers testosterone concentrations is unclear. Some studies have shown no effect of spironolactone on testosterone levels in cis men or in the treatment of cis women with hirsutism [9, 10]. One report demonstrated that spironolactone may impair the achievement of goal serum estradiol in transgender women [6]. In contrast, the Endocrine Society Guidelines cite a small study from 1989 supporting the notion that spironolactone lowers testosterone levels [1, 7]. This study reported a greater fall in testosterone after 12 months of therapy with spironolactone 200-600 mg/day and a cyclic hormone regimen that included medroxyprogesterone acetate (MPA), compared to high-dose estrogen therapy alone [7]. Major limitations of this study included the lack of differentiation between the effect of spironolactone versus MPA, very high doses of spironolactone, and the inability to measure estrogen concentrations because they used conjugated equine estrogen. A more recent study found testosterone levels reliably in the female range in a quarter of gender-incongruent patients treated with spironolactone and estrogen but did not find a correlation between spironolactone dose and testosterone suppression [11]. Furthermore, low doses of estrogen were used, with a median estradiol level of only 33 to 99 pg/mL, and there was no estrogen-only comparison group [11]. Not surprisingly, many patients did not reach goal testosterone levels.

Regardless of insufficient evidence-based efficacy studies, anecdotal benefit to maximum anti-androgen therapy is not infrequent. Spironolactone also blocks the action of adrenal androgens and could be useful, in particular, for androgen-mediated hair loss. A risk-benefit discussion acknowledging our limited understanding is warranted with all patients initiating hormone therapy.

Progestogens 

Many patients inquire about progestogen use given anecdotal reports of improved breast development, mood, and/or libido. Currently, there is insufficient data to recommend for or against progestogens. Given concerns about the risk of breast cancer, atherosclerotic cardiovascular disease, and VTE in post-menopausal cis women on MPA, randomized controlled trials (RCTs) are desperately needed to clarify potential benefits [12]. Bio-identical micronized progesterone may have a lower risk of breast cancer and VTE compared to synthetic progestins such as MPA and might be preferable if a benefit can be established.

Testosterone monitoring 

The Endocrine Society Guidelines recommend a goal testosterone level below 50 ng/dL for AMAB individuals on estrogen therapy [1]. However, this does not take into consideration that estrogens increase sex hormone-binding globulin (SHBG), resulting in higher total testosterone levels despite lower free and bioavailable testosterone. One study showed that 33% of transgender individuals on estrogen for six months had total testosterone levels above the cis female range, but only 7% had an increased free testosterone [13]. It is important to consider checking free testosterone, bioavailable testosterone, and/or SHBG to better assess the degree of testosterone suppression. It is also important to keep in mind that since some anti-androgens inhibit testosterone action rather than production, testosterone levels do not necessarily correlate with the degree of androgen inhibition.

Individualized approach 

Gender is a spectrum and transition goals vary. While some people seek maximal feminization, others wish to attain certain permanent changes, such as breast growth, and then stop hormones. Other individuals may forgo hormone therapy completely. Some patients may be seeking de-androgenization rather than feminization, with factors such as the prevention of scalp hair loss playing a significant role. Patient goals for sexual activity and sexual function are also diverse [14]. Some individuals prefer to suppress erectile function maximally, while others hope to maintain it. Given this, we need to understand how using anti-androgens in AMAB individuals with testosterone at the lower end of the cis-female range could affect libido and erectile function. We need to understand the relationship between serum hormone concentrations and clinical outcomes and be mindful that not all individuals desire a total testosterone level below 50 ng/dL. While the UCSF guidelines have updated their management recommendations to accommodate an individualized approach, other guidelines need to expand their recommendations to match the diverse goals of the community [2].

Gender-affirming hormone management over the lifespan is another area requiring further attention in clinical guidelines. The Endocrine Society Guidelines recommend titrating estrogen to achieve an estradiol level of 100-200 pg/mL, which is derived from the average estradiol concentration of menstruating cis women [1]. We believe that goal estradiol levels should vary based on patient-specific goals and different stages of transition. During the first two years of therapy, or the induction phase, when physical changes are most rapid, it might be reasonable for goal estradiol levels to be closer to 200 pg/mL. While some studies using lower-dose estrogen have not found a correlation between estradiol level and physical changes, RCTs with a range of dosages are needed to answer this question [15]. After producing adequate physical changes, a maintenance phase with lower estradiol goals while maintaining testosterone suppression may optimize lifetime estrogen exposure. This may be particularly true if the patient has received a gonadectomy. Notably, without gonadectomy, a moderate concentration of estradiol remains necessary to maintain testosterone suppression. However, if gonadectomy is performed, the maintenance estradiol dose can be significantly lowered as long as sufficient estrogen is present for bone health. 

There is some evidence that prolonged use of estradiol can lead to testicular atrophy and that maintenance doses of estrogen could potentially be lowered to reduce possible adverse effects even if gonadectomy is not performed; however, studies on the effect of GAHT on testicular tissue are inconsistent, likely due to variability in GAHT regimens [16]. Patients undergoing estrogen reduction should monitor androgen levels and phenotypic change.

Conclusion 

While research into GAHT for gender-incongruent AMAB individuals has increased, substantial knowledge gaps remain. Providers need active-control RCTs assessing discrete, patient-centered clinical outcomes to better meet the community’s diverse goals. Clinical guidelines should be updated to reconcile discrepancies in estrogen dosing and provide a more individualized approach. 
